# Cadmium Exposure Disrupts Periodontal Bone in Experimental Animals: Implications for Periodontal Disease in Humans

**DOI:** 10.3390/toxics6020032

**Published:** 2018-06-13

**Authors:** Andrew W. Browar, Emily B. Koufos, Yifan Wei, Landon L. Leavitt, Walter C. Prozialeck, Joshua R. Edwards

**Affiliations:** 1 College of Dental Medicine, Midwestern University, Illinois, 555 W. 31st St., Science Hall, 211-J, Downers Grove, IL 60515, USA; ekoufos39@midwestern.edu (E.B.K.); ywei89@midwestern.edu (Y.W.); lleavitt83@midwestern.edu (L.L.L.); 2 Department of Pharmacology, Midwestern University, Downers Grove, IL 60515, USA; wprozi@midwestern.edu (W.C.P.); jedwar@midwestern.edu (J.R.E.)

**Keywords:** cadmium, periodontal disease, periodontitis, alveolar bone, osteotoxicity, one health

## Abstract

Cadmium (Cd) is an environmental contaminant that damages the kidney, the liver, and bones. Some epidemiological studies showed associations between Cd exposure and periodontal disease. The purpose of this study was to examine the relationship between Cd exposure and periodontal disease in experimental animals. Male Sprague/Dawley rats were given daily subcutaneous injections of Cd (0.6 mg/kg/day) for up to 12 weeks. The animals were euthanized, and their mandibles and maxillae were evaluated for levels of periodontal bone by measuring the distance from the cementoenamel junction (CEJ) to the alveolar bone crest (ABC) of the molar roots. After 12 weeks of Cd exposure in animals, there was a significantly greater distance between the CEJ and ABC in the palatal aspect of the maxillary molars and the lingual aspect of the mandibular molars when compared with controls (*p* < 0.0001). This study shows that Cd has significant, time-dependent effects on periodontal bone in an animal model of Cd exposure. These findings support the possibility of Cd being a contributing factor to the development of periodontal disease in humans.

## 1. Introduction

The World Health Organization recognizes that chronic diseases often share common associations. For example, factors such as diet, lack of physical activity, environmental exposures, and tobacco use are associated with various health problems [1]. Chronic periodontitis is an oral disease that attacks the supporting structures of the teeth (gingiva and jaw bone). Besides affecting oral health, periodontal disease is associated with many other chronic health conditions, including cardiovascular disease and diabetes [2]. Periodontitis is an inflammatory process in response to dental plaque bacteria that activates the innate and adaptive immune responses [3]. It affects approximately 47% of the United States (US) population over 30 years old [4]. 

The use of tobacco products is a major factor in oral disease, with a dose-related and cumulative relationship with the severity of periodontitis [5]. Smoking was also shown to create a dysbiosis of the oral bacterial flora, favoring more pathogenic bacteria [6]. 

Of the many toxic components that make up tobacco smoke, the metal cadmium (Cd) is notable in that it is a group 1 carcinogen with toxic effects in lung, liver, testicular, kidney, and bone tissues [7]. Cd is a universally present and naturally occurring environmental contaminant found in a wide variety of common types of food, such as spinach, sunflower seeds, beef liver, and peanuts [8,9]. Diet is a major source of Cd exposure, especially for people living in areas with high levels of Cd contamination [10]. In vivo, Cd exhibits complex toxicokinetics, complicating efforts to understand the mechanisms of Cd toxicity [11,12]. Cd can be absorbed from the lung or the gastrointestinal (GI) tract, before being quickly distributed to the liver where it becomes sequestered upon binding to metallothionein. However, as hepatocytes die from either Cd-induced injury, or general cell turnover, the Cd can redistribute to other tissues, especially the kidney, but also to pancreas and bone. As a result of its tendency to be sequestered in tissues, the half-life for the elimination of Cd from the body is estimated to be as high as 30 years [13]. It is also important to note that blood levels of Cd are usually only elevated during acute, relatively high-level exposure. As Cd accumulates in tissues, blood levels tend to fall. Little or no cadmium is excreted in the urine until the epithelial cells of the proximal tubule are injured by Cd [11,12]. The kidney is considered the primary target organ of Cd toxicity, with concentrations reaching the highest levels in the renal cortex over time. While Cd is known to cause hypercalciuria via renal injury, and lead to osteoporosis via indirect means, Cd also has direct osteotoxic effects on bone tissue, resulting in enhanced bone resorption [14,15]. Biomarkers for bone formation and resorption, such as serum osteocalcin and urinary cross-linked N-telopeptide of type I collagen, were significantly correlated with Cd exposure in a population in Thailand with high dietary Cd intake [16]. Cd having direct and indirect effects on bone formation is significant to the current study because women with osteoporosis are more likely to exhibit periodontal bone loss [17], a hallmark of periodontitis. Cd exposure is also associated with diabetes mellitus and altered metabolic hormone homeostasis [9].

Blood and urinary Cd levels are associated with periodontal disease in the US [18] and South Korea [19,20]. Actual tooth Cd content was higher in a group of patients with periodontal disease [21]. However, other studies did not find significant associations between blood Cd levels and periodontal disease in South Korea [22] or Poland [23]. In addition, the statistical analysis and methodology used by Arora et al. [18] to conclude that a link exists between Cd and periodontal disease were called into question [24]. 

Because the literature shows contradictory results as to whether Cd exposure is associated with periodontal disease, the goal of the current study was to determine if Cd affects periodontal alveolar bone in a well-established animal model of chronic Cd exposure in rats. 

## 2. Materials and Methods

### 2.1. Animal Studies

The jaw samples used in the presented study were harvested from rats that were treated with Cd in a series of studies of renal toxicity and urinary biomarkers [25]. All animal studies were conducted in compliance with the United Sates National Institutes of Health (NIH) Guide for the Care and Use of Laboratory Animals (National Research Council of National Academies 2011), and were approved by the Institutional Animal Care and Use Committee of Midwestern University. Adult male Sprague/Dawley rats weighing 250–300 g (Envigo, Indianapolis, IN, USA) were housed socially (two rats per plastic cage) on a 12 h/12 h light/dark cycle. Animals in the Cd treatment group (N = 5–10) received daily (Monday–Friday) subcutaneous injections of CdCl_2_ at a dose of 0.6 mg (5.36 µmol)/kg in 0.24–0.35 mL isotonic saline for up to 12 weeks. Control group animals (N = 5–10) received daily injections of the saline vehicle only. Animals were euthanized at 6, 9, and 12 weeks, and the tissues were harvested.

### 2.2. Collection and Preparation of Jaw Samples

Jaws were harvested after animals were anesthetized with an intraperitoneal injection of ketamine/xylazine (67/7 mg/kg) and the kidneys removed. The carcasses were decapitated, and then, the jaws were harvested. Dental scissors (HuFriedy SCGCP) were used to cut through the mandibular symphysis along the floor of the oral cavity lateral to the base of the tongue, and then, through the ramus of the mandible. Buccal musculature and soft tissue were dissected by cutting through the fornix of the buccal vestibule anteriorly. A similar dissection was carried out on the opposite side. The maxillae were harvested by cutting through the palate posterior to the maxillary molars, continuing anteriorly through the fornix of the maxillary vestibule, cutting through the zygomatic process, and across the pre-maxilla through the maxillary incisor teeth. The maxillae were then separated into halves by cutting through the mid-palatal suture. The jaw samples were then placed in labeled containers, and stored in a −80 °C freezer. 

Frozen jaw samples (N = 5–10 per treatment group at each time point) were brought to room temperature, defleshed by boiling in water for 7 min to 10 min, manually debrided of soft tissue with periodontal instruments, and then soaked overnight in 5% sodium hypochlorite. The following day, the samples were again carefully debrided of any remaining soft tissue, rinsed, and placed in 3% hydrogen peroxide overnight. Afterward, they were again cleaned, rinsed, and then stained with 1% methylene blue for 1 min, before being rinsed and dried to demarcate the cementoenamel junction (CEJ).

### 2.3. Morphometric Analysis of Periodontal Bone Levels

Rat jaw segments were affixed to glass microscope slides using soft wax, and viewed using a Nikon E400 microscope with a 2× objective lens, and digital images were captured using an Evolution MP digital air-cooled color camera with the Image Pro Plus image acquisition software (Version 6.1, MediaCybernetics, Rockville, MD, USA, 2006).

**Figure 1:**
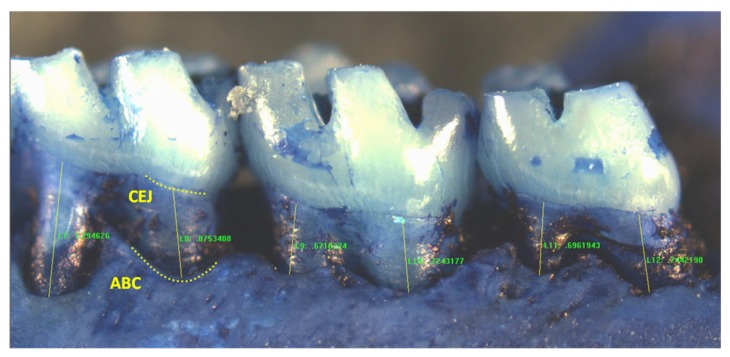
Representative image of a mandibular right lingual molar segment showing measurements from the cementoenamel junction (CEJ) to the alveolar bone crest (ABC) in a rat molar sample. (Mesial measure of first molar not shown).

To quantify periodontal bone levels, Image Pro Plus image analysis software was utilized. The distance from the CEJ to the alveolar bone crest (ABC) was measured along the main body of each root of each molar (Figure 1). For each molar segment, three values for each first molar root were recorded, and two for each second and third molar (total of seven for each segment). Measurements were taken from the right and left maxillary buccal, maxillary palatal, and mandibular lingual molar segments (six segments per animal).

### 2.4. Statistical Analysis

Data were analyzed using the Graph Pad Prism statistical program (Version 6.1, La Jolla, CA, USA, 2006). Mean values of the distance from the CEJ to the ABC (mm) for each molar root were evaluated for the saline control versus the Cd-treated animals at the six-week and 12-week time points, using a two-way analysis of variance (ANOVA). If significant differences were detected, a post-hoc Tukey’s test was then used to compare values from the time-matched control with the Cd-treated animals. Furthermore, mean measurements from the pooled maxillary buccal, maxillary palatal, and mandibular lingual measurements were also compared. For all analyses, *p* ≤ 0.05 was considered as statistically significant. 

## 3. Results

Cd was associated with a time-dependent decrease in periodontal bone levels in an animal model of long-term Cd exposure. The animals tolerated the daily subcutaneous injections of Cd very well, and no animal died or was removed from the study early due to an excessive loss (>20%) of body weight. 

Twelve-week samples included two cohorts of experimental and control animals (N = 6 + 4) whereas the six-week sample included only one cohort (N = 5), accounting for the difference in sample size (Table 1).

Mandibular buccal values were not measured because of their proximity to the external oblique ridge and the ascending ramus to the alveolar bone. Nine-week samples were not included because they were not readable. A protracted time (>9 months) in freezer storage prior to processing resulted in the teeth being loose in their alveolar housing, and measurements were not reliable (Table 1).

**Figure 2:**
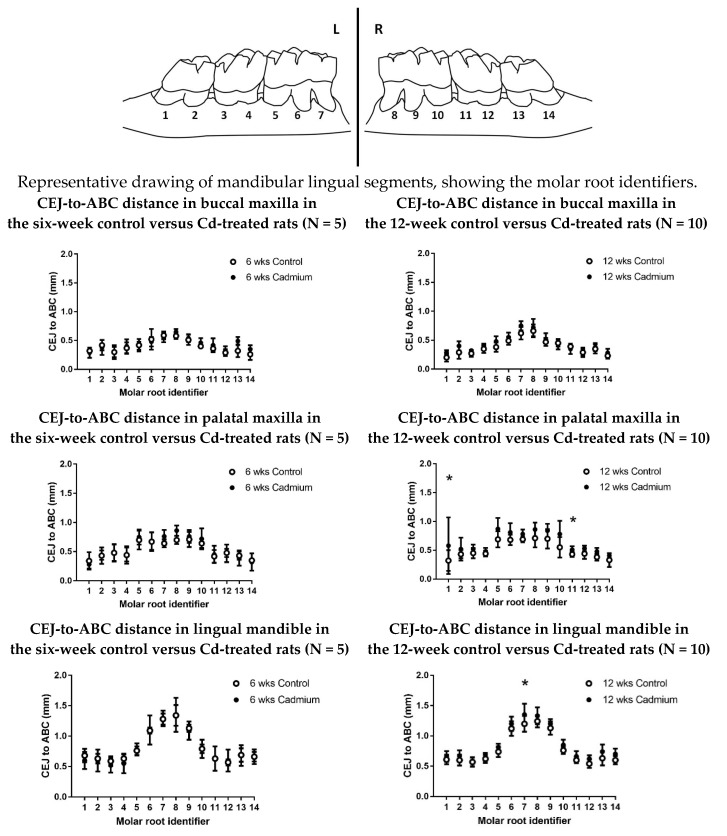
Graphs comparing the CEJ-to-ABC distances in Cd-treated (0.6 mg/kg/day) animals and saline-treated control animals for individual molar roots. An asterisk (*) indicates significant differences between matching control values, as determined by two-way analysis of variance (ANOVA), followed by Tukey’s post-hoc multiple comparison tests; data are given as mean ± SD; N = 5–10.

After 12 weeks of Cd exposure to the experimental animals, there was a significantly greater distance between the cementoenamel junction (CEJ) and the alveolar bone crest (signifying poorer periodontal bone levels) at the palatal aspect of the maxillary molars and the lingual aspect of the mandibular molars when compared with saline-treated control animals (*p* < 0.0001) (Figure 2 and Table 2). A time-dependent change was shown with the maxillary palatal aspect from a comparison of the six-week and 12-week experimental groups (*p* < 0.0001). In the mandibular lingual aspect comparison of the six-week and 12-week experimental groups, the difference was nearly significant (*p* = 0.053).

## 4. Discussion

The literature shows a number of studies relating the prevalence of periodontal disease to an exposure to environmental Cd [18,19,20,22,23], with varying methodologies and conclusions. Arora [18] used a partial-mouth method detailed in the Third National Health and Nutrition Examination Survey (NHANES III) [26]. Han [19], Won [20], and Kim [22] used a partial-mouth method from the Community Periodontal Index of Treatment Needs (CPITN) [27]. Herman [23] utilized the CPITN method; however, it was not specified whether a full-mouth or partial-mouth method was used, and their threshold for periodontitis was not identified. Furthermore, the partial-mouth methodology was shown to underestimate the prevalence of disease [28,29], and this potentially affected their outcomes in an unknown way.

This is the first published study examining the effect of Cd on periodontal changes in a controlled animal study. Previously, copper (copper sulfate) exposure in drinking water was investigated in experimental hamsters. That study showed that dietary copper improved periodontal bone levels, particularly in males [30]. 

The results presented in this study showed that animals given daily doses of Cd (0.6 mg/kg/day) for up to 12 weeks had significant, time-dependent changes of periodontal bone levels, as measured by the CEJ-to-ABC distance. This particular dosing protocol was chosen as it was widely used in studies examining the effects of cadmium on organs such as the kidney, the liver, the pancreas, and bones [12,25,31,32]. This protocol can be used to reproducibly induce various levels of cadmium toxicity, from mild levels at six weeks to severe levels at 12 weeks. In addition, the patterns of Cd distribution and toxicity with this model were comparable to those with chronic oral exposure [11]. Most importantly, since this protocol was used extensively, and is widely accepted as a standard in the Cd field [33,34,35,36], it allows for the comparison and interpretation of results across different studies. 

Several direct and indirect mechanisms may be responsible for the apparent effects of Cd on periodontal bone levels. Cd exerts direct osteotoxic effects on bone tissue, as well as having indirect effects on bone metabolism and changes in blood calcium regulatory hormones, including parathyroid hormone levels in humans [15]. Numerous studies in the experimental animal and in vitro literature show that Cd in very low doses has direct osteotoxic effects, causing osteoblasts and osteoblast precursor cells to undergo degeneration, while increasing osteoclast formation or activity [14]. In a tissue culture of mouse calvaria osteoblasts, exposure to Cd was found to stimulate a release of calcium from the tissue culture [37,38]. In an earlier study, calcium release from a similar culture was found to be mediated by the production of prostaglandin E2 [39], a potent cytokine for bone resorption. In a study of a peripheral blood mononuclear cell (PBMC) culture, low doses of Cd stimulated the expression of messenger RNA (mRNA) for interleukin 1 (IL-1) and tumor necrosis factor alpha (TNFα), and also of IL-6 at higher doses [40,41], all of which are inflammatory mediators related to periodontitis and periodontal bone loss. In a subsequent study of PBMC cultures and Cd exposure, bacterial antigens of *Salmonella enteritidis* had an immune-modulatory effect on inflammatory mediator expression [42], suggesting that a bacterial challenge coupled with Cd exposure will differentially affect an immunological response to either challenge. These same pro-inflammatory mediators are also associated with cardiovascular disease and diabetes [2].

Another potential mechanism of Cd-induced bone changes may result from Cd-induced renal injury or dysfunction, directly affecting blood calcium levels and subsequent bone metabolism [43]. The animal model of Cd exposure used in this study (0.6 mg/kg/day) was developed to examine the nephrotoxic effects of Cd, with sensitive urinary biomarkers of renal injury, such as kidney injury molecule-1 (Kim-1) and cystatin C, beginning to appear around the fourth week, followed by overt and progressive renal dysfunction starting around the ninth week [25]. It is interesting to note that the change in periodontal bone levels reported here became statistically significant after the time point when overt renal toxicity would be apparent (e.g., after the ninth week). This indicates that some of the Cd effects on the periodontium may be due to indirect effects on whole-body calcium levels due to renal dysfunction. 

There are specific aspects of Cd toxicodynamics that need to be taken into consideration when examining the studies showing [18,19,20] or not showing [22,23] associations between measures of Cd exposure (e.g., blood or urinary Cd) and periodontal disease, namely the source of the sample to determine Cd exposure. Cd is a cumulative toxin with blood or urine levels typically reflecting recent or short-term exposures. This would suggest that the Cd effects on the periodontium occur over time. Elevated urinary Cd levels may also be the result of proximal tubule epithelial cell death, and are not necessarily due to recent exposures, especially in older individuals [12].

When comparing experimental animal models of Cd poisoning and human exposures, it is also important to consider the sources of Cd exposure. Some authors convincingly argued that an overlooked source of human Cd exposure, as related to periodontal disease, is the use of intraoral dental alloys [44]. Human teeth were shown to be a site of Cd accumulation, and presumably, a source of exposure to adjacent alveolar bone. In a study of both smokers and non-smokers with periodontal disease, tooth Cd levels were ten-fold or higher when compared with teeth from patients who did not have periodontal disease [21]. Therefore, the possibility exists that Cd is absorbed into teeth from dental restorations or tobacco smoke, and may have direct effects on neighboring periodontal bone.

This study is currently limited to male Sprague/Dawley rats. Morphometric studies investigating periodontal bone changes with other experimental studies (not involving Cd) showed a gender difference in responses [30,45], and Cd also showed gender-specific changes in bone responses in humans [16]. Future studies should examine the effects of Cd on periodontal bone loss in both male and female animals. 

There was a significantly greater distance between the CEJ and the ABC (signifying poorer periodontal bone levels) when comparing the 12-week Cd-exposed animals with the saline-treated controls (*p* < 0.0001) at the palatal aspect of the maxillary molars, and the lingual aspect of the mandibular molars. This was not observed after six weeks (Table 2). It is worth noting that bone levels for the 12-week maxillary buccal Cd-treated animals did not reach a level of statistical significance when compared with controls. The anatomy of the periodontal bone at the alveolar crest is somewhat thicker on the buccal aspect of the maxilla than in the other sites, and may explain the results reported here. This difference between buccal and palatal or lingual changes was reported in other morphometric experimental studies (not involving Cd) in rats and mice [45,46,47]. The pattern of bone changes in the six-week and 12-week experimental groups were remarkably uniform, with a consistent level of bone crest for each tooth and/or root, and no significant changes in interproximal bone levels were observed. (e.g., see Figure 2 diagram between root identifiers 2–3, 4–5, 10–11, and 12–13). When comparing the maxillary palatal six-week to 12-week experimental groups, there was a significant difference (*p* < 0.0001). When comparing the mandibular lingual six-week to 12-week experimental groups, the difference was nearly significant (*p* = 0.053). As we replicate the study and increase the N values, we anticipate that the data will show more significant differences (Table 2).

While the results in this study show the effects of experimental Cd exposure on periodontal bone levels, the findings do not address the etiology of Cd-induced periodontal bone changes. Further study is needed to identify the mechanism(s) responsible for Cd-mediated periodontal bone loss, as well as the effects age, gender, systemic disease (e.g., diabetes and cardiovascular disease), and local factors (e.g., bacterial challenge, smoking, or toxicity from dental restorations) have on the Cd–periodontium interaction. 

## Figures and Tables

**Table 1:** Animals examined at each time point.

Treatment	Week 6	Week 9	Week 12a	Week 12b
N control	5	5 *	6	4 **
N experimental	5	5 *	6	4 **

* Due to a processing error these samples were not analyzed. ** Cohort contained six rats to start. Two were harvested for a histologic study.

**Table 2:** Mean measurements from pooled maxillary buccal, maxillary palatal, and mandibular lingual values.

Segment	Treatment	6-Week Mean (mm) N = 5 Per Group	SD	12-Week Mean (mm) N = 10 Per Group	SD
Maxilla/Buccal	Control	0.404	±0.134	0.398	±0.151
	Cd-treated	0.431	±0.127	0.441	±0.168
Maxilla/Palatal	Control	0.531	±0.171	0.527	±0.180
	Cd-treated	0.558	±0.216	0.645 * *p* < 0.0001, # *p* < 0.0001	±0.235
Mandible/Lingual	Control	0.820	±0.273	0.785	±0.269
	Cd-treated	0.803	±0.316	0.858 * *p* < 0.0001, # *p* = 0.053	±0.290

All values the distance in mm from the cementoenamel junction (CEJ) to the alveolar bone crest (ABC). * The *p*-value comparing the same week-matched control with the Cd-treated sample. # The *p*-value comparing the six-week sample with the 12-week sample of the same treatment.

## References

[1] World Health Organization Facing the Facts #1, Chronic Diseases and Their Common Risk Factors. http://www.who.int/chp/chronic_disease_report/media/Factsheet1.pdf.

[2] Burt B. (2005). Position paper: Epidemiology of periodontal diseases. J. Periodontol..

[3] Cekici A., Kantarci A., Hasturk H., Van Dyke T.E. (2014). Inflammatory and immune pathways in the pathogenesis of periodontal disease. Periodontology 2000.

[4] Eke P.I., Dye B.A., Wei L., Thornton-Evans G.O., Genco R.J. (2012). Prevalence of periodontitis in adults in the United States: 2009 and 2010. J. Dent. Res..

[5] Tomar S.L., Asma S. (2000). Smoking-attributable periodontitis in the United States: Findings from NHANES III. J. Periodontol..

[6] Borojevic T. (2012). Smoking and periodontal disease. Mater. Sociomed..

[7] Jarup L., Berglund M., Elinder C.G., Nordberg G., Vahter M. (1998). Health effects of cadmium exposure—A review of the literature and a risk estimate. Scand. J. Work Environ. Health.

[8] Paschal D.C., Burt V., Caudill S.P., Gunter E.W., Pirkle J.L., Sampson E.J., Miller D.T., Jackson R.J. (2000). Exposure of the U.S. population aged 6 years and older to cadmium: 1988–1994. Arch. Environ. Contam. Toxicol..

[9] Edwards J., Ackerman C. (2016). A review of diabetes mellitus and exposure to the environmental toxicant cadmium with an emphasis on likely mechanisms of action. Curr. Diabetes Rev..

[10] Satarug S., Swaddiwudhipong W., Ruangyuttikarn W., Nishijo M., Ruiz P. (2013). Modeling cadmium exposures in low- and high-exposure areas in Thailand. Environ. Health Perspect..

[11] Prozialeck W.C., Edwards J.R. (2010). Early biomarkers of cadmium exposure and nephrotoxicity. Biometals.

[12] Prozialeck W.C., Edwards J.R. (2012). Mechanisms of cadmium-induced proximal tubule injury: New insights with implications for biomonitoring and therapeutic interventions. J. Pharmacol. Exp. Ther..

[13] Friberg L. (1984). Cadmium and the kidney. Environ. Health Perspect..

[14] Bhattacharyya M.H. (2009). Cadmium osteotoxicity in experimental animals: Mechanisms and relationship to human exposures. Toxicol. Appl. Pharmacol..

[15] Schutte R., Nawrot T.S., Richart T., Thijs L., Vanderschueren D., Kuznetsova T., Van Hecke E., Roels H.A., Staessen J.A. (2008). Bone resorption and environmental exposure to cadmium in women: A population study. Environ. Health Perspect..

[16] Nishijo M., Nambunmee K., Suvagandha D., Swaddiwudhipong W., Ruangyuttikarn W., Nishino Y. (2017). Gender-specific impact of cadmium exposure on bone metabolism in older people living in a cadmium-polluted area in Thailand. Int. J. Environ. Res. Public Health.

[17] Penoni D.C., Fidalgo T.K., Torres S.R., Varela V.M., Masterson D., Leao A.T., Maia L.C. (2017). Bone density and clinical periodontal attachment in postmenopausal women: A systematic review and meta-analysis. J. Dent. Res..

[18] Arora M., Weuve J., Schwartz J., Wright R.O. (2009). Association of environmental cadmium exposure with periodontal disease in U.S. adults. Environ. Health Perspect..

[19] Han D.H., Lee H.J., Lim S. (2013). Smoking induced heavy metals and periodontitis: Findings from the Korean National Health and Nutrition Examination Surveys 2008–2010. J. Clin. Periodontol..

[20] Won Y.S., Kim J.H., Kim Y.S., Bae K.H. (2013). Association of internal exposure of cadmium and lead with periodontal disease: A study of the fourth Korean National Health and Nutrition Examination Survey. J. Clin. Periodontol..

[21] Alhasmi A.M., Gondal M.A., Nasr M.M., Shafik S., Habibullah Y.B. (2015). Detection of toxic elements using laser-induced breakdown spectroscopy in smokers’ and nonsmokers’ teeth and investigation of periodontal parameters. Appl. Opt..

[22] Kim Y., Lee B.K. (2013). Association between blood lead and mercury levels and periodontitis in the Korean general population: Analysis of the 2008–2009 Korean national health and nutrition examination survey data. Int. Arch. Occup. Environ. Health.

[23] Herman M., Golasik M., Piekoszewski W., Walas S., Napierala M., Wyganowska-Swiatkowska M., Kurhanska-Flisykowska A., Wozniak A., Florek E. (2016). Essential and toxic metals in oral fluid—A potential role in the diagnosis of periodontal diseases. Biol. Trace Elem. Res..

[24] Dye B.A., Dillon C.F. (2010). Elevated cadmium exposure may be associated with periodontal bone loss. J. Evid. Based Dent. Pract..

[25] Prozialeck W.C., VanDreel A., Ackerman C.D., Stock I., Papaeliou A., Yasmine C., Wilson K., Lamar P.C., Sears V.L., Gasiorowski J.Z. (2016). Evaluation of cystatin C as an early biomarker of cadmium nephrotoxicity in the rat. Biometals.

[26] Albandar J.M., Brunelle J.A., Kingman A. (1999). Destructive periodontal disease in adults 30 years of age and older in the United States, 1988–1994. J. Periodontol..

[27] Ainamo J., Barmes D., Beagrie G., Cutress T., Martin J., Sardo-Infirri J. (1982). Development of the World Health Organization (WHO) Community Periodontal Index of Treatment needs (CPITN). Int. Dent. J..

[28] Eke P.I., Thornton-Evans G.O., Wei L., Borgnakke W.S., Dye B.A. (2010). Accuracy of NHANES periodontal examination protocols. J. Dent. Res..

[29] Miller N.A., Benamghar L., Roland E., Martin G., Penaud J. (1990). An analysis of the community periodontal index of treatment needs. Studies on adults in France. III—Partial examinations versus full-mouth examinations. Community Dent. Health.

[30] Costich E.R. (1955). A Quantitative Evaluation of the Effect of Copper on Alveolar Bone Loss in the Syrian Hamster. J. Periodontol..

[31] Prozialeck W.C., Vaidya V.S., Liu J., Waalkes M.P., Edwards J.R., Lamar P.C., Bernard A.M., Dumont X., Bonventre J.V. (2007). Kidney injury molecule-1 is an early biomarker of cadmium nephrotoxicity. Kidney Int..

[32] Prozialeck W.C., Edwards J.R., Lamar P.C., Liu J., Vaidya V.S., Bonventre J.V. (2009). Expression of kidney injury molecule-1 (Kim-1) in relation to necrosis and apoptosis during the early stages of Cd-induced proximal tubule injury. Toxicol. Appl. Pharmacol..

[33] Aoyagi T., Hayakawa K., Miyaji K., Ishikawa H., Hata M. (2003). Cadmium nephrotoxicity and evacuation from the body in a rat modeled subchronic intoxication. Int. J. Urol..

[34] Dudley R.E., Gammal L.M., Klaassen C.D. (1985). Cadmium-induced hepatic and renal injury in chronically exposed rats: Likely role of hepatic cadmium-metallothionein in nephrotoxicity. Toxicol. Appl. Pharmacol..

[35] Goyer R.A., Miller C.R., Zhu S.Y., Victery W. (1989). Non-metallothionein-bound cadmium in the pathogenesis of cadmium nephrotoxicity in the rat. Toxicol. Appl. Pharmacol..

[36] Shaikh Z.A., Northup J.B., Vestergaard P. (1999). Dependence of cadmium-metallothionein nephrotoxicity on glutathione. J. Toxicol. Environ. Health A.

[37] Carlsson L., Lundholm C.E. (1996). Characterisation of the effects of cadmium on the release of calcium and on the activity of some enzymes from neonatal mouse calvaria in culture. Comp. Biochem. Physiol. C Pharmacol. Toxicol. Endocrinol..

[38] Romare A., Lundholm C.E. (1999). Cadmium-induced calcium release and prostaglandin E2 production in neonatal mouse calvaria are dependent on COX-2 induction and protein kinase C activation. Arch. Toxicol..

[39] Suzuki Y., Morita I., Yamane Y., Murota S. (1989). Cadmium stimulates prostaglandin E2 production and bone resorption in cultured fetal mouse calvaria. Biochem. Biophys. Res. Commun..

[40] Marth E., Barth S., Jelovcan S. (2000). Influence of cadmium on the immune system. Description of stimulating reactions. Cent. Eur. J. Public Health.

[41] Marth E., Jelovcan S., Kleinhappl B., Gutschi A., Barth S. (2001). The effect of heavy metals on the immune system at low concentrations. Int. J. Occup. Med. Environ. Health.

[42] Hemdan N.Y., Emmrich F., Sack U., Wichmann G., Lehmann J., Adham K., Lehmann I. (2006). The in vitro immune modulation by cadmium depends on the way of cell activation. Toxicology.

[43] Horiguchi H., Oguma E., Sasaki S., Miyamoto K., Ikeda Y., Machida M., Kayama F. (2005). Environmental exposure to cadmium at a level insufficient to induce renal tubular dysfunction does not affect bone density among female Japanese farmers. Environ. Res..

[44] Guzzi G., Pigatto P.D., Ronchi A. (2009). Periodontal disease and environmental cadmium exposure. Environ. Health Perspect..

[45] Rivaldo E.G., Padilha D.P., Hugo F.N. (2005). Alveolar bone loss and aging: A model for the study in mice. J. Periodontol..

[46] Abe T., Hajishengallis G. (2013). Optimization of the ligature-induced periodontitis model in mice. J. Immunol. Methods.

[47] Crawford J.M., Taubman M.A., Smith D.J. (1978). The natural history of periodontal bone loss in germfree and gnotobiotic rats infected with periodontopathic microorganisms. J. Periodontal Res..

